# The intersection of land use and human behavior as risk factors for zoonotic pathogen exposure in Laikipia County, Kenya

**DOI:** 10.1371/journal.pntd.0009143

**Published:** 2021-02-19

**Authors:** Joseph Kamau, Elizabeth Ashby, Lindsey Shields, Jennifer Yu, Suzan Murray, Megan Vodzak, Allan Ole Kwallah, Peris Ambala, Dawn Zimmerman

**Affiliations:** 1 Institute of Primate Research, Nairobi, Kenya; 2 Department of Environmental Science and Policy, George Mason University, Fairfax, Virginia, United States of America; 3 PATH, Washington, DC, United States of America; 4 Global Health Program, Smithsonian Conservation Biology Institute, Smithsonian Institution, Washington, DC, United States of America; 5 Kenya Medical Research Institute, Nairobi, Kenya; 6 Department of Biochemistry, Kenyatta University, Nairobi, Kenya; Oregon State University College of Veterinary Medicine, UNITED STATES

## Abstract

A majority of emerging infectious diseases (EIDs) are zoonotic, mainly caused through spillover events linked to human-animal interactions. We conducted a survey-based human behavioral study in Laikipia County, Kenya, which is characterized by a dynamic human-wildlife-livestock interface. Questionnaires that assessed human-animal interactions, sanitation, and illnesses experienced within the past year were distributed to 327 participants among five communities in Laikipia. This study aimed to 1) describe variation in reported high-risk behaviors by community type and 2) assess the relationship between specific behaviors and self-reported illnesses. Behavioral trends were assessed in R via Fisher’s exact tests. A generalized linear mixed model with Lasso penalization (GLMMLasso) was used to assess correlations between behaviors and participants’ self-reported illness within the past year, with reported behaviors as independent variables and reported priority symptoms as the outcome. Reported behaviors varied significantly among the study communities. Participants from one community (Pastoralist-1) were significantly more likely to report eating a sick animal in the past year (p< 0.001), collecting an animal found dead to sell in the past year (p<0.0001), and not having a designated location for human waste (p<0.0001) when compared to participants from other communities. The GLMMLasso revealed that reports of an ill person in the household in the past year was significantly associated with self-reported illness. Sixty-eight percent of participants reported that bushmeat is available within the communities. Our study demonstrates community-level variation in behaviors that may influence zoonotic pathogen exposure. We further recommend development of targeted studies that explore behavioral variations among land use systems in animal production contexts.

## Author summary

Many infectious diseases, such as H1N1, Ebola, and COVID-19, can be spread to humas from animals. In order to reduce the risk of disease “spillover” (disease transmission from an animal to a person), it is important to understand how interactions between humans and animals can influence spread. Certain behaviors, such as eating raw meat, hunting, or sharing drinking water with animals can put people at greater risk of contacting bacteria and viruses that can cause these diseases. Globally, communities that depend on animal production are at heightened risk due to increased contact with animals. In this study, the authors conducted human behavioral surveys among different communities in Kenya that raise livestock. Results show that reported behaviors varied greatly by community. One of the communities reported a significantly higher proportion of behaviors, such as eating raw meat or eating animals found dead. Communities that show high prevalence of these behaviors may be at greater risk for contracting diseases from animals. Understanding this variation is important for developing plans for community outreach and addressing behaviors that can influence risk of disease spread.

## Introduction

Emerging Infectious Diseases (EIDs) can be defined as infectious diseases that have appeared in a novel population, are geographically expanding, or are appearing with increased incidence [1,2]. Of particular concern are zoonotic diseases, which constitute over 60% of EIDs [3], suggesting a significant risk at the human-animal interface. While the link between human-animal contact and disease transmission is widely reported, understanding specific human behaviors at local levels is integral to identifying potential mechanisms of zoonotic disease emergence [4,5].

In Kenya, resource sharing, or common use of grazing and watering resources among humans and animals, is prevalent in pastoral communities and within arid and semi-arid land (ASAL) areas [6,7]. Human interactions with domestic animals can increase risk of zoonosis transmission within communities that practice animal production, particularly via contact with infected food and water [4,8,9,10]. A 2015 study identified Rift Valley Fever (RVF), brucellosis, Q fever, and influenza-like illnesses among ten priority zoonoses in Kenya based on their severity of illness, pandemic potential, and socioeconomic impact [11]. Each of these diseases is endemic to Kenya and has been reported annually in humans and animals within multiple counties [11]. RVF and brucellosis are known to affect both domestic (cattle, sheep, goats, camels) and wildlife species (buffalo, antelope) [8,12,13]. Behaviors such as consuming raw animal products and consuming or handling products from sick animals are known transmission pathways for several of these priority pathogens [12,13]. During a 2006–2007 RVF outbreak that affected six Kenyan provinces, individuals who reported these behaviors were more likely to experience acute cases [12]. Meanwhile, brucellosis is endemic in Kenya but underreported due to its nonspecific febrile symptoms in humans and lack of reliable diagnostic testing [4,13]. For Brucellosis, RVF, and Q-fever, human infections are most commonly associated with livestock interactions [5,8,12,13]. Though these pathogens are also known to infect wildlife, additional research is required to understand transmission mechanisms between wild and domestic species [8,13]. RVF and brucellosis are two of the primary diseases that threaten both the lives and livelihoods of communities that depend on livestock production, particularly in ASALs in Kenya [4,12].

Human interactions with wildlife can likewise present opportunities for disease transmission. Behaviors of greatest concern include consumption of bushmeat, which may put humans at risk of anthrax, brucellosis, and bovine tuberculosis, among others [14,15]. Humans are especially vulnerable to exposure when slaughtering, butchering, or consuming raw meat from wild animals [16]. Other human-wildlife interactions can also lead to disease spillover. Human cases of Marburg virus in Kenya have previously been associated with visits to caves that house infected bats [17]. The wildlife-livestock interface also plays a role in human risk by facilitating pathogen spread among wild and domestic species. Previous studies in Kenya suggest that resource sharing among wildlife and livestock may transmit RVF virus and *Brucella* spp., thus facilitating cycles of endemism, though more research is needed to understand these linkages and subsequent risks to human populations [18]. Understanding localized human-animal interactions and behaviors involving wildlife and livestock is essential for identifying risk factors and potential mechanisms for disease transmission [4,15].

Laikipia County, located in Kenya’s Rift Valley province, was selected as the study site due to its dynamic human-wildlife-livestock interface. With animal production as its largest economic activity, livestock populations in Laikipia have rapidly increased over recent decades [19]. In addition, Laikipia is one of only two counties in Kenya whose wildlife populations have increased [20]. Wildlife and livestock commonly share land and water sources in limited resource settings, such as ASALs like Laikipia County [21,22]. As livestock and wildlife populations continue to expand, this may enhance opportunities for transmission of pathogens between species. This carries implications for human risk, as interactions with livestock may also expose humans to diseases originating from wildlife [23]. Wildlife-livestock interactions therefore contextualize the ecological drivers of zoonosis spillover.

The complex human-wildlife-livestock interface in Laikipia County, Kenya leads to a heightened risk of zoonotic disease emergence. To date, there is a dearth of studies that examine human interactions with both wildlife and livestock in regard to disease risk in Kenya. To address this gap, we conducted a survey-based study in five communities in Laikipia to investigate human behaviors related to livestock management, food and water practices, sanitation, and wildlife interaction that may influence risk of exposure to zoonotic pathogens. In addition, the survey addressed livestock veterinary care and management practices to better understand how livestock health may impact human health. In this study, we aimed to 1) assess variation in reported behaviors that can influence zoonotic disease exposure risk among five communities in Laikipia, 2) identify behaviors (as outlined in aim 1) and demographic variables that correlate to self-reported illnesses, and 3) evaluate the variation in livestock veterinary care and management among the communities. These data can inform targeted intervention measures to address risk of pathogen exposure on a local level.

## Methods

### Ethics statement

The survey tool and research protocol were reviewed and approved by the IRB board of the University of California-Davis (IRB ID # 804522–32) and the Kenya Medical Research Institute in Nairobi. Verbal consent was obtained from each participant before proceeding with the interview, as permitted by the IRB. Children from age 12–17 were included if an adult was present and gave consent.

### Site characterization

**Fig 1:**
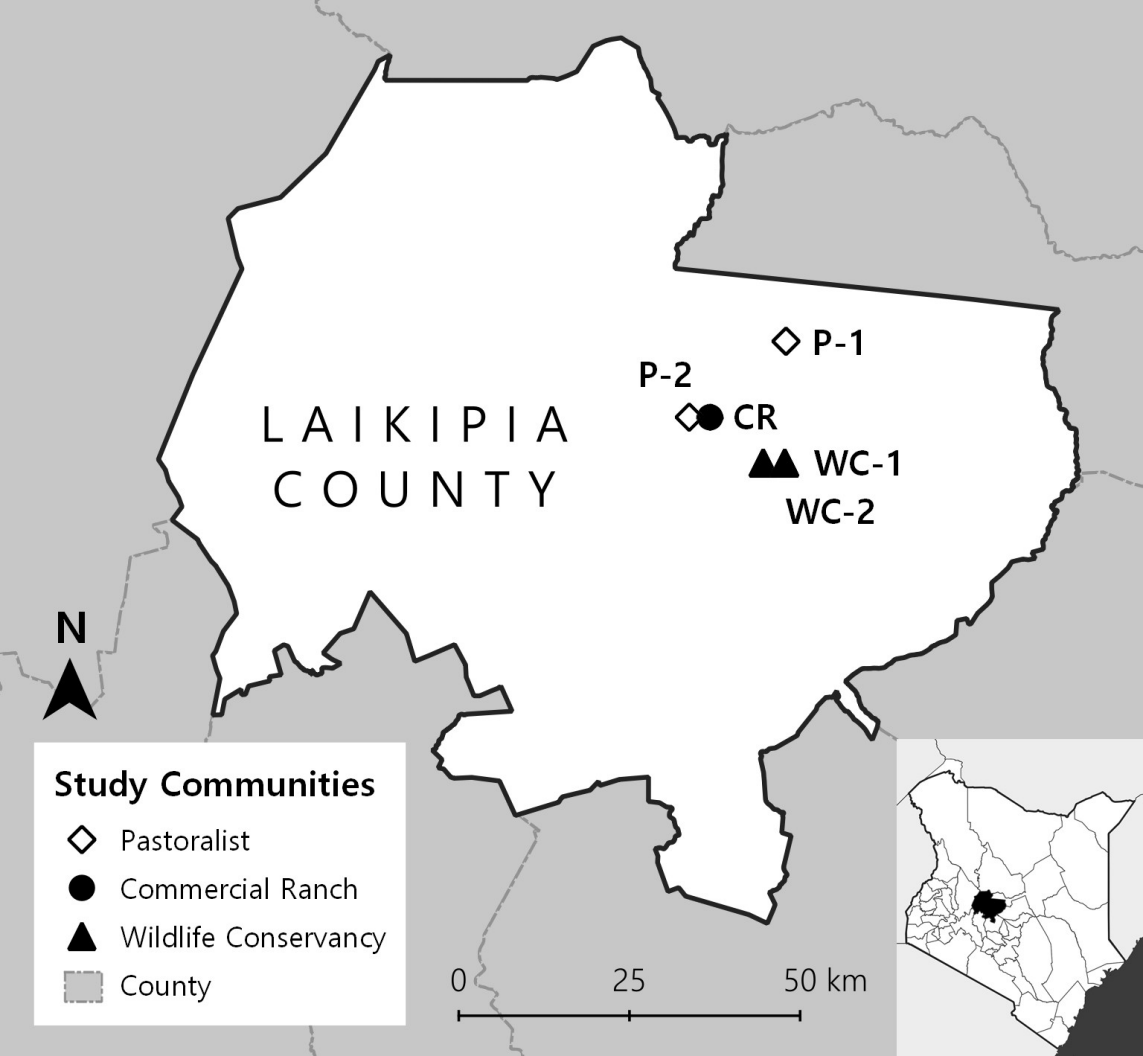
Distribution of study communities in Laikipia County, Kenya. Data sources: Natural Earth. OpenAfrica, http://africaopendata.org, CC BY 4.0. Map created in QGIS 3.16.13 [27].

Surveys were conducted in five townships within Laikipia County (located in the Laikipia North sub-county): Pastoralist-1 (P1), Pastoralist-2 (P2), Commercial Ranching (CR), Wildlife Conservancy-1 (WC-1), and Wildlife Conservancy-2 (WC-2) (Fig 1). Animal production is a primary economic activity in each location, although characteristics of land use vary. Previous studies have identified pastoralism, commercial ranching, and wildlife conservancies as primary land use systems in Laikipia [24,25,26]. Pastoralism is a subsistence-based animal production strategy in which livestock keepers move herds through collectively managed areas [24]. Commercial ranches differ in that individuals often own land and produce animal goods for sale [25]. Wildlife conservancies preserve land for wildlife use while incorporating tourism activities and some livestock production activities [25]. Local experts identified the land use strategy employed by each community and provided estimates for human and livestock populations (Table 1). WC-1 and WC-2 are located within a wildlife conservancy. P-2 and CR are located outside the northwest boundary of this same wildlife conservancy. Residents of P-1 and P-2 retrieve water directly from nearby rivers or dams. CR, WC-1, and WC-2 residents have water piped into the communities.

**Table 1:** Land use type, human and livestock population estimates by community.

		Community				
		P-1	P-2	CR	WC-1	WC-2
Land Use Type		Pastoralist	Pastoralist	Commercial Ranching	Wildlife Conservancy	Wildlife Conservancy
Human Population Est.		5,000	1,000	350	350	350
Livestock Population Est.	Cattle	4,000	400	400	None	
	Sheep/Goats	10,000	6,000	2,000	1,000	
	Camels	None	None	90	None	

* Livestock estimates were given for both WC-1 and WC-2 collectively.

### Study design and data collection

Individuals in each township were surveyed during two rounds of surveys conducted in September 2017 and May 2018, using a structured questionnaire (S1 Data). Each community included in this project had associated Community Health Volunteers (CHVs), who recruited survey participants and served as liaisons with researchers. Prior to the study, CHVs held community-wide meetings in their respective towns to describe the purpose of the project. CHVs instructed willing participants to meet at a designated location within the community (school, clinic) on the day of the survey. All willing participants present at the designated location on the predetermined days were included. Participants received compensation that was considered appropriate and culturally relevant by the local PREDICT research teams, including food or household goods valued at less than 10 USD. Surveys were conducted in a private location between a single participant and either a CHV or Kenyan PREDICT researcher administering the survey. Prior to conducting the study, all CHVs received human subjects research training from Collaborative Institutional Training Initiative (CITI), as well as PREDICT training for questionnaire administration. A consent form was read aloud, and participants gave verbal consent prior to beginning the survey. Questions were read aloud in the participant’s preferred language, and the researcher recorded the verbal responses on paper. All participants responded to the primary questionnaire (“General Survey”, S1 Survey), with questions addressing human-animal interactions (both livestock and wildlife), sanitation, and disease perception. Participants also reported demographics, such as age, sex, education, place of residence, and family size. Participants who identified “animal production” as their primary occupation completed an additional supplemental questionnaire (“Animal Production Survey”) with questions pertaining to animal management, sanitation practices, and diseases affecting livestock. Following collection, data were transferred to CSV files and uploaded to a secure online repository. Based on population estimates from CHVs (Table 1), the participation rates by community are as follows: 2% of P-1, 10% of P-2, 15% of CR, 15% of WC-1, and 5% of WC-2. The sample size from each community is shown in Table 2.

**Table 2:** Participant demographics by community.

Gender	Age Group (Child < 18, Adult ≥ 18)	P-1 n = 102	P-2 n = 103	CR n = 53	WC-1 n = 51	WC-2 n = 18
Male	Adult	39	19	30	20	9
	Child	1	10	4	0	0
Female	Adult	56	56	16	29	9
	Child	5	18	3	2	0

Demographic distribution of human behavioral questionnaire participants in Laikipia County, Kenya. *P = Pastoralist*, *CR = Commercial Ranching*, *WC = Wildlife Conservancy*.

* Gender of one participant was listed as “other”, and was excluded from gender analysis.

Participants were asked if they had experienced an illness in the past year that they considered to be “unusual”. A follow-up open-ended question allowed participants to describe symptoms they experienced during this “unusual illness”. The survey contained a list of grouped symptoms that may be indicative of priority illnesses (Table 3). These symptoms were not read aloud to participants, but were present for the researcher’s reference. Researchers noted the symptoms for each illness event that the participant reported. If all of the symptoms associated with a priority illness were reported for a single illness event, then the researcher marked which priority illness corresponded to the participant’s report. Symptoms that did not correspond to priority illness, including singular symptoms, were recorded but not noted as a “priority illness”.

**Table 3:** Lists of symptoms and potential illnesses as listed in the general module.

Symptoms reported (for the same reported illness event)	Priority Illness
Fever with headache and severe fatigue or weakness	Encephalitis
Fever with bleeding or bruising not related to injury	Hemorrhagic fever
Fever with cough and shortness of breath or difficulty breathing	Severe Acute Respiratory Illness (SARI)
Fever with muscle aches, cough, or sore throat	Influenza-Like Illness (ILI)

### Data analysis

Quantitative analysis of the behavioral questionnaire responses was conducted in R 3.5.2 [28]. Answers of “don’t know” given in response to a behavioral question were coded as “no” for analysis. All behavior variables were binary “yes or no” responses. Fisher’s exact tests with a Holm p-value adjustment were conducted using the fmsb package to compare frequencies of each of the behavioral variables that related to animal contact and sanitation across the five communities (significance at p<0.05) [29]. Interactions with specific animal species were omitted if there was not sufficient variation in responses. For this reason, we only report variation in interaction with camels, cattle, cats, dogs, and wild ungulates. Fewer than ten participants reported interactions with other wildlife species, and fewer than ten participants reported no interaction with poultry or sheep/goats.

A binary yes/no response variable of “priority symptoms” was created to indicate if each participant reported symptoms corresponding to any of the priority illnesses (Table 3). A generalized linear mixed model with Lasso penalization (GLMMLasso) with a binomial link function was then used to test associations between behavioral and demographic variables with the outcome variable of self-reported “priority symptoms” in the past year. This method combines the Lasso regression, which shrinks low parameter coefficients to 0, with a GLMM; this provides variable selection while allowing input of both fixed and random factors [30]. Analysis was conducted with the glmmLasso package [31]. To build the initial model, demographic variables and all behavioral variables from the survey that related to sanitation, indirect, or direct contact with wildlife or livestock (including waste and products) were included (see S1 Table for the categorization of each variable). Each variable included is generally understood to facilitate human contact with zoonotic pathogens. Only species interactions with camels, cattle, cats, and dogs were included in the model. Behavioral and demographic variables were set as fixed effects, while community was coded as a random effect. The penalty parameter was selected by comparing BIC values of model outputs within a range of 1:100. As the goals of the study are to identify 1) behaviors reported in each community and 2) variables associated with self-reported illness across the sample, incorporating “community” as a random effect adequately accounts for spatial autocorrelation. Any further spatial autocorrelation would not affect the interpretation of results (e.g., frequency of behaviors reported in each community).

A majority of the behavioral questions on the survey related to food and water consumption practices. To conduct a more targeted analysis, two GLMM models were constructed using the lme4 R package that contained 1) all food-related and 2) all water-related behaviors [32]. Both models contained a binomial link function. “Community” was set as a random effect, with “priority symptoms” as the outcome variable. QQ residual plots were assessed using the DHARMa package in R [33] and revealed normal distributions for each of the models. Descriptive statistics were also conducted to evaluate livestock care practices for the Animal Production Survey.

## Results

### General survey

#### Behaviors and exposure risk

A total of 327 participants responded to the general module among the five study locations (Table 2). Participants reported a variety of primary occupations, which were categorized as animal production (n = 119), migrant laborer (n = 52) student (n = 41), housework (n = 24), and crop production (n = 9). The remaining 82 participants reported “other” primary occupations, such as sales, construction, and teaching.

Table 4 shows percentages of participants who reported behaviors that may increase risk of exposure to pathogens and variation in reported behaviors among communities. Of P-1 participants, a majority reported eating sick animals (98%), collecting animals found dead to eat (95%) or sell (94%), and not treating drinking water (81%). P-1 was significantly less likely to report a designated location for human waste when compared to all other communities (p < 0.0001). All participants in P-2 reported sharing a drinking water source with animals, while 55% reported not treating drinking water. P-1 was significantly more likely to report certain high-risk behaviors when compared to P-2 (eating raw meat or blood, collecting dead animals to sell: p < 0.0001). In WC-1, 43% reported sharing a drinking water source with animals and 86% did not treat drinking water, compared to 11% and 61% for these behaviors in WC-2.

**Table 4:** Variation in frequency of reported behaviors by community.

	Number of respondents per site (%)				
	P-1 *n = 102*	P-2 *n = 103*	CR *n = 53*	WC-1 *n = 51*	WC-2 *n = 18*
**Water from uncovered source**	101 (99)^A^	100 (97) ^A^	5 (9) ^B^	0 (0) ^B^	3 (17) ^B^
**Drinking water untreated**	83 (81) ^A^	57 (55) ^B^	22 (42) ^B^	44 (86)^A^	11 (61) ^A, B^
**Drinking water source used by animals**	92 (90) ^A^	103 (100) ^B^	12 (23) ^C^	22 (43) ^C^	2 (11) ^C^
**Eaten raw/undercooked meat in past year**	100 (98)^A^	51 (49) ^B^	30 (57) ^B^	3 (6) ^C^	1 (6) ^C^
**Eaten Sick animal in past year**	100 (98) ^A^	57 (55) ^B^	24 (45) ^B^	1 (2) ^C^	0 (0) ^C^
Found dead animal, collected to eat or share in past year	97 (95) ^A^	52 (50) ^B^	16 (30) ^C^	5 (10) ^D^	0 (0) ^D^
Found dead animal, collected to sell in past year	96 (94) ^A^	13 (13) ^B^	6 (11) ^B^	1 (2) ^B^	0 (0) ^B^
No designated location for human waste	57 (56)^A^	13 (13)^B^	2 (4)^B^	10 (20) ^B^	1 (6) ^B^

Results of Fisher’s exact test, which compared the frequency of reported behaviors across all five communities. Percentages are indicated in parentheses. Each question elicited a binary “yes/no” response. Groups that do not share a letter are significantly different from each other (p<0.05).

GLMMLasso was run with an optimal L-1 penalty parameter of 20 (see S1 Table for the full list of variables in the GLMMLasso). The response variable of “priority symptoms reported in the past year” was a binary yes/no variable. Community was included as a random effect. “Age” and “number of people in household” were continuous variables. “Occupation” was coded as a factor with five levels. All other fixed effects were binary categorical variables. The only factor returned with a nonzero coefficient was participant reports of an “ill person in the household in the past year” (coefficient est. 1.62). While 72 out of the 327 participants reported having an “unusual illness” in the past year, only 49 participants reported symptoms that were considered “priority symptoms”.

Two GLMMs were constructed to assess the relative influence of food and water consumption behaviors on the outcome variable (participant-reported “priority symptoms”). There were no significant associations between any single variable with reported symptoms (Table 5).

**Table 5:** Comparison of GLMMs associated with food and water consumption behaviors.

Model	Variables	Intercept	z-value	p-value	Confidence Interval (2.5–97.5%)
**Water consumption behaviors**	Treat drinking water	-0.62	-1.70	0.09	(-1.4) - 0.07
	Share drinking water source with animals	0.87	1.78	0.07	(-0.07)– 1.9
	Obtain water from uncovered source	-0.47	-1.12	0.25	(-1.3)– 0.4
**Food consumption behaviors**	Animal feces found in food	-0.001	-0.002	0.9	(-0.8) - 0.8
	Ate raw meat	-0.17	-0.32	0.7	(-2.6)–(-1.3)
	Collected dead animal to eat	0.68	1.45	0.15	(-0.2) -1.6
	Ate sick animal	-0.18	-0.32	0.75	(-1.3) - 0.9

GLMM models that contain variables related to food and water consumption behaviors. The outcome variable for both models was participant’s self-reported “priority symptoms”, which was coded as a binary variable (yes/no). Community was included as a random effect in both models. All fixed effects were binary (yes/no) categorical variables. The ‘yes’ response was the reference level for each fixed effect.

#### Knowledge and attitudes

When asked if worried about disease outbreaks among animals at local markets, 225 of the 327 participants responded “yes” (69%). Regarding risk to slaughtering or butchering with an open wound, 44 (13%) made an association to risk of disease infection. Respondents most frequently reported that the behavior was risky, but did not know what the risks were (n = 131, 40%). There was no significant difference in self-reported illness among participants who acknowledged a risk, versus participants who did not (p = 1) (Table 6).

**Table 6:** Responses related to knowledge and perceptions.

		Total (n = 327)	P-1 (n = 102)	P-2 (n = 103)	CR (n = 53)	WC-1 (n = 51)	WC-2 (n = 18)
Worried about disease outbreak among livestock in markets?	Yes	225 (69%)	56 (55%)	89(86%)	51 (96%)	21 (41%)	8 (44%)
	No	102 (31%)	46 (45%)	14 (14%)	2 (4%)	30 (59%)	10 (56%)
Are there any risks to slaughtering/butchering when you have an open wound?	Yes	233 (71%)	69 (68%)	82 (80%)	47 (89%)	27 (53%)	8 (44%)
	No	38 (12%)	19 (18%)	5 (5%)	4 (7%)	8 (16%)	2 (12%)
	Don’t Know	56 (17%)	14 (14%)	16 (15%)	2 (4%)	16 (31%)	8 (44%)

* Some participants selected multiple responses.

### Animal production survey

Of the general study population, 112 also completed the Animal Production Survey (Table 7). Of the Wildlife Conservancy communities, one participant lived in WC-2, and 18 lived in WC-1. These data were compiled into a single “WC” site for this survey. P-1 and WC most frequently reported bushmeat availability on site (78% and 79%, respectively). There was no significant association between seeking veterinary care and experiencing outbreaks among livestock (p = 0.9). P-1 and P-2 were the least likely to have received veterinary care in the past year. Fifteen percent of pastoralists in P-1 reported an outbreak in animals during the past year, yet only 5% said that ill animals were quarantined or destroyed. Reports of bushmeat on site were not significantly associated with participant’s reported illness (p = 0.6).

**Table 7:** Responses to questions from the Animal Production Survey.

Animal Production Survey (n = 112)		Total	P-1 (n = 65)	P-2 (n = 18)	CR (n = 10)	WC (n = 19)
Bushmeat available on Site?	Yes	76	51 (78%)	9 (50%)	1 (10%)	15 (79%)
	No	36	14 (22%)	9 (50%)	9 (90%)	4 (21%)
Outbreak among livestock in past year?	Yes	15	10 (15%)	3 (17%)	2 (20%)	0 (0%)
	No	97	55 (85%)	15 (83%)	8 (80%)	19 (100%)
Have livestock received veterinary care in the past year?	Yes	48	14 (22%)	8 (44%)	10 (100%)	16 (84%)
	No	64	51 (78%)	10 (56%)	0 (0%)	3 (16%)
Have animals been quarantined or destroyed in the past year because of disease?	Yes	6	3 (5%)	2 (11%)	1 (10%)	0 (0%)
	No	106	62 (95%)	16 (89%)	9 (90%)	19 (100%)

## Discussion

Risk of zoonotic disease transmission is dependent on interacting ecological and human behavioral factors. This study contributes to a growing body of research that aims to assess behaviors and perceptions influencing risk of disease spillover within a complex human-wildlife-livestock interface. The objective was to compare behaviors and perceptions among communities in order to assess variation in factors that may influence zoonotic disease exposure risk.

Overall, participants reported high rates of behaviors that can increase risk of exposure to zoonotic pathogens, which varied on a community level. P-1 and P-2 were the only communities in which a majority of the population reported obtaining water from an uncovered source and sharing a drinking water source with animals while not treating drinking water. This suggests that participants in P-1 and P-2 may be at greater risk of contacting pathogens, such as *Leptospira* spp., which are known to be transmitted via water contaminated with animal waste [34]. Furthermore, lack of clean drinking water may have implications for coronavirus transmission. Studies indicate that MERS-CoV, SARS-CoV-1, and SARS-CoV-2 may be transmissible in water via the fecal-oral route [35]. Drinking water that is untreated and drawn from a source that can be contaminated by human or animal waste can result in exposure to diverse pathogens [36,37]. As indicated by local researchers, CR, WC-1, and WC-2 have water piped into the communities, which may or may not be reliably treated beforehand. Understanding the water treatment process would enhance our assessment of potential pathogen exposure from water sources. Practices surrounding food preparation and consumption likewise reveal differentiated risks in potential pathogen exposure on a community level. Behaviors such as consuming raw meat/blood, sick animals, or animals found dead have previously been associated with RVF, brucellosis, anthrax, Q fever, and leptospirosis in Kenya [4,9,12,13,16]. Combined with the observed variation in water use practices, P-1 features the greatest proportion of overall high-risk behaviors. Local researchers indicated that WC-1 and WC-2 may be wealthier than other communities, which could explain some of the variation in factors such as water accessibility. Difference in culture and lifestyle may also explain some of the behavioral variation. Pastoralist communities are centered around livestock, which are an important source of food and trade [38]. Previous studies show that animal products may compose more than 80% of the diet among some pastoral communities in Kenya [38]. Furthermore, consuming raw milk and blood is a significant source of nutrition [5,38]. These studies corroborate the data collected in this study, which detected higher rates of raw meat/blood consumption in Pastoral communities compared to Wildlife Conservancy communities.

Veterinary care access and livestock management likewise varied by community. Data reveal that animal production workers who experienced disease outbreaks did not always quarantine or destroy the affected livestock, indicating risk to herds and animal keepers. Combined with high reports of consuming sick animals, particularly in P-1, the data suggest possible transmission pathways for RVF, brucellosis, and other diseases transmitted by contact with sick animals and consumption of their products [4,12,13]. Pastoralist range lands are often remote and far-removed from veterinary services, which presents a barrier to accessing care. This factor has previously been identified as a challenge in disease surveillance among pastoralist communities [38]. Further research in this region should assess how movement and land use affect accessibility of veterinary services.

In addition to variation in these behaviors, a primary finding of this study is that bushmeat availability was widely reported in each site. Bushmeat has been linked to spillover events of Ebola and Marburg viruses, anthrax, hepatitis, and parasitic infections [15]. Not only is meat itself potentially infectious, but the process of capturing, slaughtering, and butchering wildlife poses a substantial risk for a spillover event [39]. Further studies should focus on collection and trade of bushmeat, identifying species that are most commonly purchased and sold, particularly in regions with high wildlife densities such as Laikipia. Responses to “knowledge and perceptions” questions give evidence of a general concern for disease, yet participants lacked an understanding of transmission mechanisms. Few participants who indicated awareness of risks of butchering with an open wound identified a connection to disease. This suggests that limited knowledge is still a barrier to implementing protective measures in target communities.

Previous studies have discovered variation in pathogen exposure risk based on land use type. Bett et al. [9] showed that pastoralists in drylands in Kenya express greater seroprevalence of *Brucella* spp. and *Leptospira* spp. when compared to individuals in irrigated lands. This study suggests that limited water sources in drylands may intensify resource sharing among people, livestock, and wildlife, thus increasing opportunities for transmission of *Leptospira* spp. Likewise, brucellosis is reported to have high burdens in pastoralist communities due to practices in food consumption and handling. Although mechanisms of *Brucella* spp. transmission are generally well known, there is a greater need to understand variation in local practices that can facilitate spread in order to inform public health interventions [4]. As both pathogen presence and behaviors can vary based on land use type, we recommend that these factors should be examined together in greater depth. The GLMMLasso for this study did not reveal significant associations between individual food/water practices and reported illness. However, results of the GLMM reveal a positive though non-significant association between sharing a drinking water source with animals and reported illness. Future studies should continue assessing food and water behavioral practices in the context of ASAL regions with limited water resources.

### Implications for future disease dynamics

Projections of climate change and population growth carry significant implications for disease dynamics within land use structures [40]. With the Kenyan population expected to more than double between 2016 and 2050, livestock populations in rural areas are expected to rise exponentially to meet the growing food demand [40,41]. Intensification of livestock production correlates to greater herd densities that increase risk of communicable livestock diseases and greater risk of food and water contamination [40,42]. These projections suggest an increase of land use overlap and contact among humans, wildlife, and livestock. Climate dynamics are also shifting herder preference in livestock production. From 1985–2015, camel populations in Kenya soared by 835% [19]. In previous studies, herders declared a preference for camels over traditional cattle stock, citing their resilience in increasingly long droughts [22]. Camels in Kenya have expressed high rates of seropositivity for Middle East Respiratory Syndrome (MERS) coronavirus. Though MERS is only endemic in the Middle East and an outbreak has never been detected in Kenya, MERS has been declared a priority pathogen by the World Health Organization with acknowledgement of seropositivity in camels throughout Kenya [43,44]. A recent study by Sitwa et al. found a 69% seropositivity rate among camels in Kenyan pastoral systems, indicating potential transmission risk for people in contact with camels [45]. Shifting trends in herd composition, stemming from climate and herder preference, suggests new possible risk factors [19]. Assessing species-specific disease transmission routes relies on analyzing animal movement, climate change, and pastoralist preference as related to human-animal interaction. Studies that assess human interactions with specific species and the behaviors that characterize these interactions will become increasingly relevant as land use patterns continually adjust to climate change.

### Limitations and recommendations

While the survey facilitated assessment of behavioral variation on a community level, we acknowledge some limitations from the survey structure. Self-reported “unusual” illness is a subjective outcome variable. On the survey, this question is vague and open to interpretation. Though the list of “priority symptoms” was designed to reduce this subjectivity, reported symptoms could still be characterized inaccurately. Likewise, determining behavioral risk depended on participant’s accuracy in self-reported behaviors, which may be impeded by perceived stigma or lacking memory of certain behaviors. For example, post-study community follow-ups revealed that residents of P-2 may be more likely to perform certain behaviors, such as eating raw meat or blood, than reported on the survey. In addition, hunting behavior is unlikely to be accurately reported since hunting wildlife is illegal. Bushmeat availability was highly reported, but not the action of hunting, suggesting that hunting is a common practice but underreported. This is a limitation with any survey-based study, but still offers useful insight into behavioral variation.

Many questions on the survey permitted only a “yes/no” response, which does not indicate frequency of reported behaviors. Participants responding “yes” to eating raw meat or blood in the past year could have engaged in this activity only once, or on a daily basis. Frequency of these activities is important in assessing pathogen exposure risk. Overall, survey questions were not context-specific, as they were developed for use in multiple countries, which impedes detailed analysis at a local level. Focus group discussions and semi-structured interviews would provide more context for framing disease risk, especially in regard to knowledge and attitudes. Qualitative data would also permit further analysis of behavioral variation, such as reasons why WC communities were less likely to report certain behaviors (e.g., eating sick animals) when compared to P communities.

Continuing engagements should be tailored to communities based on the most prevalent high-risk behaviors. Programs in P-1, for example, should target food safety and human-animal interactions. In general, future interventions that aim to mitigate EID threats should identify high-risk behaviors and develop targeted interventions accordingly. Globally, community health workers are regarded as cornerstones of health engagement in low and middle-income countries (LMICs), indicating that their involvement is crucial to successful interventions [46]. In this study, CHVs’ rapport and local knowledge was integral to securing community participation. Ongoing engagement strategies should continue to divert resources to provide training for CHVs. Culturally appropriate educational interventions or materials on behavioral risk factors and protective measures should continue to target communities that rely on animal production.

## Conclusion

This study aimed to elucidate patterns of high-risk behaviors in a potential EID emergence hotspot. This research can help reveal the link between human behavioral risk factors, animal interactions, and land use on a fine scale. Mitigation efforts must be implemented that address community vulnerability in regions worldwide that harbor zoonoses of global concern. By understanding the risks of disease spread before an outbreak occurs, interventions can be tailored to reduce risk of emerging threats, both globally and locally. Providing training to community health volunteers should continue to be a focus of EID projects as a means of informing at-risk communities via trusted messengers. These outreach strategies bolster global efforts to strengthen local response capacities and mitigate high-risk behaviors while being rooted in a One Health approach.

## Supporting information

S1 Data Survey responses for General Survey and Animal Production Survey. (XLSX)

S1 Survey Questions and answer choices from the general survey. (PDF)

S1 Table List of all variables included in GLMM, grouped by criteria for inclusion. (DOCX)
