# Supplementary material for: The intersection of land use and human behavior as risk factors for zoonotic pathogen exposure in Laikipia County, Kenya
Source: PLoS Negl Trop Dis. 2021 Feb 19;15(2):e0009143. doi: 10.1371/journal.pntd.0009143 (PMC7894889; doi:10.1371/journal.pntd.0009143)
Supplement: S1 Survey — (PDF) [file pntd.0009143.s002.pdf]

Human Questionnaire Form

1. Consent Form Administered & Signed ◯ yes ◯ no   Participant ID:________________________

2. Description of Interview Location - Select all that apply.
(To be completed by interviewer prior to administrative questionnaire.
Prepare and download modules in advance.)
   - ◯ Animal Production or Abattoir Site
   - ◯ Crop Production Site
   - ◯ Extractive Industry Site
   - ◯ Market or Value Chain Site
   - ◯ Temporary Settlement Site
   - ◯ Tourism Site
   - ◯ Wildlife Restaurant
   - ◯ Zoos or Sanctuaries
   - ◯ Hospital or Clinic - Health Professional
   - ◯ Hospital or Clinic - Patient
   - ◯ Protected Area (eg. forest, public park)
   - ◯ Other: ________________________

3. Date of interview ________________________

4. Begin time of interview ________________
(Example: 17:50)

5. End time of interview ________________
(Example: 19:20)

6. Where are you conducting this interview?

Village/Town/City ______________ District ______________ Province/State ______________

Latitude ________________________ Longitude ________________________
Interviewer: Please collect GPS coordinates if administering using paper and pen.

7. Interviewer Observed Gender ◯ male ◯ female ◯ other

INTERVIEW/QUESTIONNAIRE BEGINS

Demographics Section (include observation question 7)

8. How old are you? ________________
If the exact age is unknown, enter the respondent's estimated age.

9. Where do you live?

Village/Town/City ______________ District ______________ Province/State ______________

Latitude ________________________ Longitude ________________________
Interviewer: Probe for landmarks or nearest known site if area unknown.
GPS coordinates to be identified and entered after completion of interview.

10. How long have you lived there? Select one option.
   - ◯ <1 month
   - ◯ 1 month - 1 year
   - ◯ >1 - 5 years
   - ◯ >5 - 10 years
   - ◯ >10 years

11. How many other people live in the dwelling where you live? ________
Skip to question 14 if answer is 0.

12. How many in the dwelling are children less than 5 years old? ________

841-279707

13. How many in the dwelling are male? ________

14. How many rooms are there in the dwelling where you live? ________
(Do not include bathroom or kitchen)

15. Is the dwelling a permanent structure (that cannot be moved)?
Interviewer: If answer is no, complete temporary settlement questionnaire.
- ○ yes
- ○ no

16. Do you get water from:
Select all that apply.
- ○ piped in water/water taps
- ○ covered well
- ○ uncovered well/pond/river
- ○ water truck/rainwater harvest
- ○ other: ____________________

17. Do you treat your drinking water?
- ○ yes
- ○ no

18. If yes, how do you treat your water?
Select all that apply.
- ○ boil
- ○ filter
- ○ add chlorine or bleach
- ○ solar disinfection
- ○ other: ____________________

19. Is your source for drinking water ever used by animals?
- ○ yes
- ○ no

20. In your dwelling is there a dedicated location for human solid waste/excreta?
(Example: toilet, latrine, designated area)
- ○ yes
- ○ no

21. Do you have containers for storing food for the household?
Select all that apply.
- ○ yes, with covers
- ○ yes, without covers
- ○ no

842-279707

 Participant ID________________
(For reference only)

Livelihood Section

In this section, I'd like to ask you about education and the kinds of work activities that you have done since this time last year.

22. What is the highest level of education you have completed?
Select one option. (Skip for Cameroon.)
- ○ primary school
- ○ secondary school
- ○ college/university/professional
- ○ none

23. What is the highest level of education that your mother completed?
Select one option. (Skip for Cameroon.)
- ○ primary school
- ○ secondary school
- ○ college/university/professional
- ○ none

24. Since this time last year what are the activities you have done to earn your livelihood?
Select all that apply.
- ○ extraction of minerals, gas, oil, timber
- ○ crop production
- ○ wildlife restaurant business
- ○ wild/exotic animal trade/market business
- ○ rancher/farmer animal production business
- ○ meat processing, slaughterhouse, abattoir
- ○ zoo/sanctuary animal health care
- ○ protected area worker
- ○ hunter/trapper/fisher
- ○ forager/gatherer/non-timber forest product collector
- ○ migrant laborer
- ○ nurse, doctor, traditional healer, community health worker
- ○ construction
- ○ other: ______________________________

25. If more than one activity was selected, what is the activity on which you spent the most time since this time last year?*
Select one option.
- ○ extraction of minerals, gas, oil, timber
- ○ crop production
- ○ wildlife restaurant business
- ○ wild/exotic animal trade/market business
- ○ rancher/farmer animal production business
- ○ meat processing, slaughterhouse, abattoir
- ○ zoo/sanctuary animal health care
- ○ protected area worker
- ○ hunter/trapper/fisher
- ○ forager/gatherer/non-timber forest product collector
- ○ migrant laborer
- ○ nurse, doctor, traditional healer, community health worker
- ○ construction
- ○ other: ______________________________

26. Which best describes your job position?
Select one option.
- ○ manager/owner/foreman
- ○ worker
- ○ live and work at home independently (If chosen, skip to question 28)
- ○ professional
- ○ other: ____________________

27. Where do you work?

Village/Town/City ____________ District ____________ Province/State ____________

Latitude ____________________ Longitude ____________________

Interviewer: Probe for landmarks or nearest known site if area unknown.
GPS coordinates to be identified and entered after completion of interview.

843-279707

Human Questionnaire Form

Participant ID________________
(For reference only)

Medical History Section

In this section, I'm going to ask you about any illness or sickness that is not known or recognized in the community, including by medical or treatment providers.

28. Where do you usually get treatment for medical problems?
    Select all that apply.
    - ◯ clinic/health center
    - ◯ hospital
    - ◯ mobile clinic
    - ◯ community health worker
    - ◯ traditional healer
    - ◯ dispensary or pharmacy

29. Have you ever had an unusual illness with any of the following symptoms:
    Select all that apply. (READ ONLY SYMPTOMS)
    - ◯ fever with headache and severe fatigue or weakness (encephalitis)
    - ◯ fever with bleeding or bruising not related to injury (hemorrhagic fever)
    - ◯ fever with cough and shortness of breath or difficulty breathing (SARI)
    - ◯ fever with muscle aches, cough, or sore throat (ILI)
    - ◯ fever with diarrhea or vomiting
    - ◯ fever with rash
    - ◯ persistent rash or sores on skin
    - ◯ no (Skip to question 33)
    - ◯ yes but, none of these symptoms-describe: ____________________________

30. Since this time last year, have you had any of these symptoms?
    - ◯ yes
    - ◯ no (Skip to question 33)

31. If yes, which ones?
    Select all that apply.
    - ◯ fever with headache and severe fatigue or weakness (encephalitis)
    - ◯ fever with bleeding or bruising not related to injury (hemorrhagic fever)
    - ◯ fever with cough and shortness of breath or difficulty breathing (SARI)
    - ◯ fever with muscle aches, cough, or sore throat (ILI)
    - ◯ fever with diarrhea or vomiting
    - ◯ fever with rash
    - ◯ persistent rash or sores on skin
    - ◯ none of these symptoms-describe:____________________________

32. In your opinion, when you were sick, what caused this sickness?
    Select all that apply.
    - ◯ contact with sick people
    - ◯ contact with wild animals
    - ◯ contact with other animals
    - ◯ bad food or water
    - ◯ bad spirits/witchcraft
    - ◯ wound or injury
    - ◯ I don't know
    - ◯ other: ______________________

33. Since this time last year, have any of the people you lived with had any of these symptoms?
    - ◯ yes
    - ◯ no (Skip to question 36)

34. If yes, which ones?
    Select all that apply.
    - ◯ fever with headache and severe fatigue or weakness (encephalitis)
    - ◯ fever with bleeding or bruising not related to injury (hemorrhagic fever)
    - ◯ fever with cough and shortness of breath or difficulty breathing (SARI)
    - ◯ fever with muscle aches, cough, or sore throat (ILI)
    - ◯ fever with diarrhea or vomiting
    - ◯ fever with rash
    - ◯ persistent rash or sores on skin
    - ◯ none of these symptoms-describe:____________________________

35. Since this time last year, did anyone you lived with die from this illness?
    - ◯ yes
    - ◯ no

844-279707

Movement Section

In this section, I'm going to ask you about any travel you have done since this time last year.

36. Have you traveled since this time last year? ◯ yes ◯ no
If answer is no, skip to the next section.

37. Where have you traveled since this time last year? Anywhere else?
Provide details, such as name of town, nearest (or most frequent) well known place if unknown by interviewer (to be linked to GPS coordinates later)
Collect up to 6 locations.
Interviewer: Probe for landmarks or nearest known site if area unknown. GPS coordinates to be identified and entered after completion of interview.

Village/Town/City ____________ District ____________ Province/State ____________
Latitude ____________________ Longitude ____________________
Notes: ____

Village/Town/City ____________ District ____________ Province/State ____________
Latitude ____________________ Longitude ____________________
Notes: ____

Village/Town/City ____________ District ____________ Province/State ____________
Latitude ____________________ Longitude ____________________
Notes: ____

Village/Town/City ____________ District ____________ Province/State ____________
Latitude ____________________ Longitude ____________________
Notes: ____

Village/Town/City ____________ District ____________ Province/State ____________
Latitude ____________________ Longitude ____________________
Notes: ____

Village/Town/City ____________ District ____________ Province/State ____________
Latitude ____________________ Longitude ____________________
Notes: ____

If there are more than six locations check here. ◯
Do not collect additional location information.

38. Why have you traveled? Select all that apply.
- ◯ work
- ◯ visit family
- ◯ moved
- ◯ religious reasons
- ◯ holiday/vacation
- ◯ go to hospital/seek medical care
- ◯ go to market
- ◯ other: ____________

845-279707

Animal Contact Section

In this section, I'm going to ask you about the animals in your life.

If answered "no" under the "In your lifetime" column, then no answer is required under the "Since this time last year" column.

Question	In your lifetime...	Since this time last year...
39. Has an animal lived as a pet in or near your dwelling?	○ yes ○ no	○ yes ○ no
40. Have you handled live animals?	○ yes ○ no	○ yes ○ no
41. Have you raised live animals?	○ yes ○ no	○ yes ○ no
42. Have you shared a water source with animals for washing?	○ yes ○ no ○ don't know	○ yes ○ no ○ don't know
43. Have you seen animal feces in or near food before you have eaten it?	○ yes ○ no	○ yes ○ no
44. Have you eaten food after an animal has touched or damaged it? (Example: chew marks or scratches)	○ yes ○ no ○ don't know	○ yes ○ no ○ don't know
45. Do any animals come inside the dwelling where you live?	○ yes ○ no	○ yes ○ no
46. Have you cooked or handled meat, organs or blood from a recently killed animal?	○ yes ○ no	○ yes ○ no
47. Have you eaten raw or undercooked meat or organs or blood?	○ yes ○ no	○ yes ○ no
48. Have you eaten an animal that you knew was not well/sick?	○ yes ○ no ○ don't know	○ yes ○ no ○ don't know
49. Have you found a dead animal and collected it to eat or share?	○ yes ○ no	○ yes ○ no
50. Have you found a dead animal and collected it to sell it?	○ yes ○ no	○ yes ○ no
51. Have you been scratched or bitten by an animal?	○ yes ○ no	○ yes ○ no

52. The last time you were scratched, bitten or cut yourself while butchering or slaughtering, what did you do?
Select all that apply.

- ○ let someone else take over
- ○ wash wound with soap and water
- ○ rinse wound with water
- ○ bandage wound
- ○ visit doctor
- ○ nothing - kept working
- ○ never butcher or slaughter

Animal Contact Section

53. Are there any risks associated with slaughtering or butchering when you have an open wound?

Interviewer: Do not read responses.

- ○ no
- ○ yes, but I don't know what they are
- ○ yes, it can make you sick
- ○ yes, it can poison you
- ○ yes, it can infect you with a disease
- ○ don't know
- ○ other: ____________________

	In your lifetime...	Since this time last year...
54. Have you slaughtered an animal?	○ yes<br>○ no	○ yes<br>○ no
55. Have you hunted or trapped an animal?<br>(If answered "yes" to "Since this time last year" also ask hunter questionnaire)	○ yes<br>○ no	○ yes<br>○ no

56. Interviewer: Circle all headings where “yes” was answered in the "Since this time last year" questions above.
Then ask which animals/mammals for each “yes” category.

	pet (39)	handled (40)	raised (41)	feces in or near food (43)	in house (45)	cooked/ handled (46)	eaten raw/ under cooked (47)	eaten sick (48)	found dead (49/50)	scratched/ bitten (51)	slaugh-tered (54)	hunted/ trapped (55)
rodents/shrews	○	○	○	○	○	○	○	○	○	○	○	○
bats	○	○	○	○	○	○	○	○	○	○	○	○
non-human primates	○	○	○	○	○	○	○	○	○	○	○	○
birds	○	○	○	○	○	○	○	○	○	○	○	○
carnivores	○	○	○	○	○	○	○	○	○	○	○	○
ungulates	○	○	○	○	○	○	○	○	○	○	○	○
pangolins	○	○	○	○	○	○	○	○	○	○	○	○
poultry	○	○	○	○	○	○	○	○	○	○	○	○
goats/sheep	○	○	○	○	○	○	○	○	○	○	○	○
camels	○	○	○	○	○	○	○	○	○	○	○	○
swine	○	○	○	○	○	○	○	○	○	○	○	○
cattle/buffalo	○	○	○	○	○	○	○	○	○	○	○	○
dogs	○	○	○	○	○	○	○	○	○	○	○	○
cats	○	○	○	○	○	○	○	○	○	○	○	○

57. Are you worried about diseases or disease outbreaks in live animals in your local market?
- ○ yes
- ○ no

END OF MAIN QUESTIONNAIRE
