# Supplementary material for: The intersection of land use and human behavior as risk factors for zoonotic pathogen exposure in Laikipia County, Kenya
Source: PLoS Negl Trop Dis. 2021 Feb 19;15(2):e0009143. doi: 10.1371/journal.pntd.0009143 (PMC7894889; doi:10.1371/journal.pntd.0009143)
Supplement: S1 Table — (DOCX) [file pntd.0009143.s003.docx]

**S3:** Variables included in original GLMMLasso model, grouped by rationale for inclusion

| **Rationale** | **Variable** |
| --- | --- |
| Potential for direct pathogen contact | Pet in home (year) |
|  | Handled animal (year) |
|  | Raised animal (year) |
|  | Animal feces found in food (year) |
|  | Ate raw meat (year) |
|  | Ate sick animal (year) |
|  | Collected dead animal to eat (year) |
|  | Collected dead animal to sell (year) |
|  | Scratched/Bitten by animal (year) |
|  | Slaughtered animal (year) |
|  | Interaction with cattle (year) |
|  | Interaction with sheep/goats (year) |
|  | Interaction with camels (year) |
| Potential for indirect pathogen contact | Share drinking water with animals |
|  | Shared water source with animals for washing (year) |
| Sanitation | Treat drinking water |
|  | Designated human waste location |
| Household Characteristics | Ill member in household (year) |
|  | Number of people in home |
| Demographics | Age |
|  | Sex |
|  | Land use type |
|  | Occupation |
|  | Community (*random effect) |

All variables entered into the original GLMMLasso model. All variables were bivariate and categorical except for Age (numeric), Number of people in home (numeric), Land use type (categorical, 3 levels), Occupation (categorical, 5 levels), and Community (categorical, 5 levels).
